# Management of children and young people (CYP) with asthma: a clinical audit report

**DOI:** 10.1038/s41533-018-0087-5

**Published:** 2018-05-21

**Authors:** Mark L Levy, Angela Ward, Sara Nelson

**Affiliations:** 1 Respiratory Clinical Lead, Harrow Clinical Commissioning Group, Harrow, Middlesex HA1 3AW UK; 2 Interim Programme Director for Models of Care, Harrow Clinical Commissioning Group Harrow, Middlesex, Harrow, HA1 3AW UK; 3 Queens Nurse: Programme Lead Children and Young People, Healthy London Partnership, Governing Body Nurse Bromley Clinical Commissioning Group, Ferguson House, London, NW1 5JD UK

## Abstract

An asthma attack or exacerbation signals treatment failure. Most attacks are preventable and failure to recognize risk of asthma attacks are well recognized as risk factors for future attacks and even death. Of the 19 recommendations made by the United Kingdom National Review of Asthma Deaths (NRAD) (1) only one has been partially implemented—a National Asthma Audit; however, this hasn’t reported yet. The Harrow Clinical Commissioning Group (CCG) in London implemented a clinical asthma audit on 291 children and young people aged under 19 years (CYP) who had been treated for asthma attacks in 2016. This was funded as a Local Incentive Scheme (LIS) aimed at improving quality health care delivery. Two years after the publication of the NRAD report it is surprising that risks for future attacks were not recognized, that few patients were assessed objectively during attacks and only 10% of attacks were followed up within 2 days. However, it is encouraging that CYP hospital admissions following the audit were reduced by 16%, with clear benefit for patients, their families and the local health economy. This audit has provided an example of how clinicians can focus learning on patients who have had asthma attacks and utilize these events as a catalyst for active reflection in particular on modifiable risk factors. Through identification of these risks and active optimization of management, preventable asthma attacks could become ‘never events’.

## Asthma attacks: learning from experience

Encouraging self-reflection and best practice can help doctors improve patient care following asthma attacks. A recent UK national review uncovered multiple shortfalls in asthma management. Mark Levy at Harrow Clinical Commissioning Group in London and co-workers conducted a clinical asthma audit to assess the after-care of 291 patients under the age of 19 who had been treated for an asthma attack in Harrow during 2016. Analysis of patient records showed that risks of future attacks were not routinely highlighted during follow-up consultations. Only a third of patients had a personal asthma action plan and only 10 per cent of attacks were followed up within two days. During the audit, doctors from each surgery attended focus groups aimed at encouraging best practice. Following the audit, patient hospital admissions from these surgeries dropped by 16 per cent.

## Introduction

The United Kingdom (UK) National Review of Asthma Deaths (NRAD)^1,2^ demonstrated major deficiencies in the management of asthma in the UK. All death certificates which included the word asthma during 2012 (*n* = 3544) were considered and notes were reviewed for over 740 of the cases classified as asthma deaths using the World Health Organization Algorythm for ICD-10 coding.^3^ Of 276 cases evaluated in a confidential enquiry; 195 (70%) were confirmed asthma deaths and over 60% had potentially preventable features. In particular the review highlighted a need for clinicians (and patients) to recognize risk of future asthma attacks; one well recognized risk being a previous asthma attack. NRAD found that 10% of the confirmed asthma deaths occurred within four weeks of hospital discharge following treatment for an acute attack, and over one fifth occurred in people who had attended Accident and Emergency departments at least once for acute asthma in the previous year.

In England, UK, the National Health Service (NHS) provision of health care is structured to include Clinical Commissioning Groups (CCGs)^4^ who commission (and pay for) NHS locally for which they are responsible. In England all general practice (GP) practices now belong to a CCG; CCGs also include other health professionals, including nurses and pharmacists. Services CCGs commission (or co-commission with NHS England) include: GP care, most planned hospital care, rehabilitative care, urgent and emergency care (including out-of-hours), most community health services, mental health and learning disability services.

In 2016/2017 there were 34 General Practices in the Harrow CCG, caring for a population of about 216,000 people (approx. 62000 < 19), with a doctor diagnosed asthma population of 11200 across all ages; with average asthma prevalence per practice of 5.2%; (2.9–10.1%); compared with the national (England) average asthma prevalence of 5.9%,^5^

With the exception of hospital admissions for acute asthma, it is difficult to get accurate data to quantify activity and costs of emergency care for patients with asthma. Hospital admission data for England is available in different formats, i.e., ICD-10 coded and Health Care Resource Group (HRG) coded data, both derived from Secondary Care Uses (SUS) data which incorporates detailed coding for services provided and is used for healthcare planning and national tarrif reimbursement. Each practice in Harrow has on average 4 and 10 acute admissions per year for asthma for children and young people (CYP) and all ages respectively using the ICD-10 coded data; the figures are 14 and 28 respectively using HRG coding for purposes of payments, all via the SUS data.^6^ Accident and Emergency attendances are broadly coded, for example, all respiratory consultations have one single code and therefore unreliable for specific disease care planning; similarly Urgent Care Centres (UCCs) caring for patients out of hours use variable codes (mainly symptoms or process) for their attendances for asthma exacerbations. In the case of general practices, the quality of coding varies considerably.

CCG performance in England, is measured by NHS England on quality health care delivery of specific areas (called Quality Premiums). Where the CCG is reported below their peer average i.e. the England average, they have to set targets and have a plan in place to achieve those targets. Harrow CCG decided to develop a quality asthma Local Incentive Scheme (LIS) with the following aims:

To develop a framework that will allow any pathway/specialty to be reviewed at any given time that would contribute to:

- Reducing variation at practice level
- Improving peer working to facilitate and enable practitioners to discuss clinical approaches and activity variances using practice based audits
- Sharing best practice and implementing agreed best practice protocols at practice level
- Reducing avoidable hospital admissions
- Developing local care pathways

This paper describes the methodology and outcomes of an asthma clinical audit, in general practices in Harrow, London, United Kingdom, and is designed to identify baseline characteristics and stimulate changes in clinical management of asthma in CYP aged under 19 years to include a follow up audit.

## Results

An asthma attack or exacerbation is a signal that treatment has failed, and we assumed that, as in the case of asthma deaths in the UK^1^ asthma attacks are potentially preventable. We therefore focused on designing a clinical audit of management before during and after asthma attacks in CYP. The main educational aim of the audit was to help clinicians, by analyzing records of CYP who were treated for attacks, increase their awareness of modifiable risk factors and to recognize that most attacks could be prevented if risks are identified and care optimized.

**Fig. 1:**
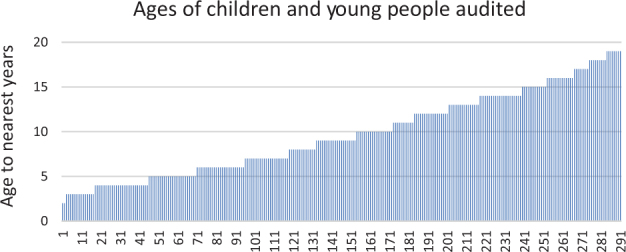
Age distribution of the children and young people audited: 291 had 333 asthma attacks

Twenty nine of the 34 Harrow general practices (85%) signed up and fully engaged in the scheme, and submitted data for the initial baseline audit (*n* = 291 patients). Individual reports with recommendations for urgent action for particular (pseudonomised) patients, were sent to all practices and subsequently discussed with a summary report for all Harrow Practices at small peer group meetings. At least one doctor representing each practice attended one of six peer group meetings where overall recommendations for Harrow were discussed. Two practices subsequently submitted data on 19 and 4 patients respectively who had suffered attacks after the end of the initial audit period (Fig. 1).

The results detailing summarized level of achievement of the standards for all 29 practices are summarized in Table 2. These results illustrate that many of these patients’ attacks may have been prevented through previous optimization of their care. Ninety two of the 158 CYPs prescribed Inhaled Corticosteroids before the attack, collected less than 4 of these inhalers in the previous year; and only a third of the 291 who had attacks, had previously been issued with a personal asthma action plan. Furthermore, management of attacks and subsequent follow up was not according to the UK guideline recommendations: during attacks oxygen saturation was measured in less than 50% and PEF was measured in less than 40%; very few patients had oxygen saturation or PEF measured after the attacks; and only 10% of the attacks were reviewed within 2 working days. (See Tables 1 and 2)

**Table 1:** Results extracted from the medical records against standards set for the Harrow CCG audit of care of 291 children and young people under 19 years old (CYP) treated for asthma attacks in 2016

Agreed audit standards	Results (evidence extracted from medical records)	
Management before attacks	*n*/total patients (%)	Notes
>6 SABA^a^ inhalers prescribed in the previous year	45/291 (15%)	Range 1–24 SABAs prescribed (>12 in 21 patients)
Evidence of issued Personal Asthma Action Plan	99/291 (34%)	
Evidence of recorded Best PEF^b^ (if >5 yrs old)	98 /221 (44%)	
Evidence of assessment of inhaler technique	73/291 (25%)	
Management during attacks	*n*/total attacks (%)	
Evidence of SaO2^c^ measurement	165 /333 (49%)—before treatment	Measured after treatment in 28 attacks
Evidence of PEF measurement (if >5 yrs old)	88/221 (39%)—before treatment	Measured after treatment in eight attacks
Evidence of short course of oral corticosteroid prescription	188/333 (56%)	Prescriptions for oral corticosteroids ‘until the attack resolved’ as in the UK guideline^d^ were provided for only two attacks; the rest were for a fixed duration of 3, 5 or 7 days
Management post attack	*n*/total attacks (%)	
Reviewed post attack	127/333 (38%)	
Reviewed within 2 working days	32/127 (25%)	Timing of reviews ranged from 1–380 days post attack

^a^SABA = Short Acting Bronchodilator Reliever (e.g., salbutamol)

^b^PEF = Peak Expiratory Flow

^c^SaO2 = oxygen saturation measured with a pulse oximeter

^d^BTS/SIGN Guideline 153^9^

**Table 2:** Detailed key messages and recommendations for practices following the Harrow audit

Key messages from the Harrow audit	Suggested actions and changes in management
An asthma attack is a sign of failed treatment this should not happen: One of the major lessons of the NRAD was that insufficient preventers (inhaled corticosteroids were prescribed in those who died. *In the Harrow Audit of asthma attacks: 92 of the 158 CYPs prescribed Inhaled Corticosteroids were prescribed less than 4 inhalers in the previous year*.	• Post attack review (include confirmation of diagnosis) with optimization of treatment within 2 working days • Strongly consider prescribing inhaled corticosteroids for all who have had an attack (see BTS/SIGN 153 guideline). • See Tables 9 and 10 in the new BTS/ SIGN Guideline for details of low, medium and high dose inhaled corticosteroids
Risk was not recognized in many of those who died from asthma in the NRAD. *In the Harrow audit, reviews were done after only 127 of the 333 attacks in CYP (32 within the 2 days recommended in BTS/SIGN)*	Assess risk when reviewing asthma patients (Table 11 SIGN/BTS; and Chapter 2—Tables 2–2 www.GINASTHMA.org)
Another major lesson from the National Review of Asthma Deaths was that excessive numbers of reliever inhalers were prescribed for those who died. *In the Harrow Asthma Audit, 45 (15%—i.e., one in six) of those CYP who had attacks were prescribed more than 6 SABA inhalers in the year before their attack. Furthermore, only 176 of 291 (58%) of prescription instructions for salbutamol read ‘when necessary’ (rest read, BD, TDS or QDS.*	• Instructions for SABA prescriptions for people with asthma should read for eg -‘Take one or two puffs for cough, wheeze or shortness of breath, and get medical help if this doesn’t help or if the relief lasts less than 4 h’ • Never prescribe salbutamol in asthma as bd, tds, or qds • Consider taking SABAs off repeat prescription or set maximum to 6 a year (HOWEVER be flexible if patients 'run out’—issue prescription and recall)
All patients with asthma should have a Personal Asthma Action Plan (PAAP). *In Harrow audit only a third of those having an asthma attack had previously been provided with a Plan****.***	• All patients with asthma should have a personal asthma action plan. See www.asthma.org or www.consultmarklevy.com→academic→lectures for examples.
**T**he BTS/SIGN guidelines for asthma attack management includes measurement of PEF & Oxygen Sats, and also that oral corticosteroids should be continued until the attack is resolved. *In the Harrow audit, oxygen saturation was measured in less than 50% of attacks and PEF was measured in less than 40% of attacks and oral corticosteroids were prescribed for 64% of the attacks. Very few patients had saturation or PEF measured after the attacks*	• Always measure oxygen saturation and peak flow when assessing a patient with uncontrolled asthma, and ideally check again after treatment to assess Rx effect; and • Always prescribe enough oral corticosteroid tablets so the attack can be treated until resolved (i.e., not just 3 or 5 or 7 days treatment). An attack = resolved when the PEF returns to usual best, and there is no need for rescue salbutamol
The SIGN/BTS Guideline for asthma states that all patients should be reviewed within 2 working days after treatment of an attack. *In the Harrow Audit, only 122 of the 333 attacks were followed up, and only 32 of these (21%) were reviewed within 2 working days.*	Consider keeping one appointment free every day for ‘acute asthma follow up’—this could be used for another patient if not taken up.

Examples of individualized feedback provided for practices are included in Appendix 2 online. Only two practices submitted subsequent (prospective) audit data for patients who had attacks after the initial audit period. Results are included for one of these in Appendix 3 online for the baseline audit (33 patients had 49 attacks) compared with the subsequent audit of 19 patients each having one attack.

Following the audit, specific recommendations were generated for Harrow practices, which are detailed in Table 2:

Data derived by the Harrow CCG using ICD-10 coded discharge data form NHS England:^6^ Harrow CCG practices achieved a 16% reduction of asthma admissions for 0–18 year olds from 189.4 per 100,000 in 2015–2016, to 159.2 per 100000 in 2016–2017.

## Discussion

We assessed the management of asthma attacks in a sample of CYP cared for by GP Practices in the Harrow CCG, North London, and made recommendations for changed practice as a result of the findings. Following the NRAD, which was led by the first author of this paper, we decided, in Harrow, to try and identify ways in which we could reduce the incidence of asthma attacks and therefore admissions to hospital for children and young people. We focused on children and young people, mainly because of the NRAD finding that care was deemed to be satisfactory in only one of the 28 CYP deaths reviewed and also due to the numbers of CYP admitted to hospital for acute asthma attacks in Harrow.^1^

### Main findings

Two years after the publication of the NRAD report ‘Why asthma still kills’ it is surprising that this audit found that: only a third of the patients had recorded evidence of being provided with a personal asthma action plan; and that risks for future attacks were not recognized—15% of CYP in Harrow treated for asthma attacks had been prescribed excessive numbers of SABA reliever inhalers in the previous year. Furthermore few patients were assessed objectively using oxygen saturation and lung function tests during attacks. Since 1992 the UK asthma guidelines have advocated a post attack review within 2 working days—to address two issues: is the attack over? and what went wrong? Despite this longstanding advice, only 10% of attacks were followed up within 2 days. However, it is encouraging that CYP hospital admissions in the year following the audit were reduced by 16%, with clear benefit for patients, their families and the local health economy.

### Strengths and limitations of this study

This audit generated data on actual treatment of 291 CYP patients who had 333 asthma attacks in 2016 in Harrow. The process did involve a time commitment by the practice staff, in that they had to identify subjects and manually extract information. Clearly this could have been simplified by an automated extraction process however the quality of data would depend on the level to which these were coded appropriately; however we believe based on informal feedback from colleagues that the process of personally extracting and reflecting on data was educationally beneficial. While difficult to quantify, this had the potential for stimulating reflective practice; which was evident at least in the case of the one practice (see appendix 3), that re-audited their care in 19 subsequent attacks demonstrating improved quality of care in our view. While these results demonstrate marked improvement in this practice, we acknowledge that, it is very likely that those who chose to participate in a second round audit are not a representative sample of practices.

Audit of medical records with reflection by clinicians has been utilized as an educational tool for many years. However some may criticize this as a valid system as it relies on health care professionals scrutinizing their own records, and self reporting limits the validity of reported data and also because missing information doesn’t necessarily mean that care was not provided or that investigations were not done. As in any study or audit, missing data in medical records is a problem; however when reflecting on their work, clinicians are able to learn the value of maintaining good, complete medical records for continuity of care.

The audit utilized standards for asthma care, based on evidence of known risks for future attacks (listed in Table 3), as a benchmark against the contents of the medical records preceding, during and after attacks. The participants were therefore able to identify preventable risk factors preceding the attacks; for example failure to provide education on recognizing attacks, by agreeing personal asthma action plans, failure to recognize poor control, through excess prescription and use of SABAs, and failure to identify whether patients can use their inhaler, by checking inhaler technique. Through discussion with colleagues, and provision of recommendations derived from the aggregate data the clinicians had an opportunity to compare and change their provision of asthma care. This was an incentivised local scheme and it is not known whether the learning from participation will continue to demonstrate benefit.

**Table 3:** Standards for the audit of children and young people (CYP) in Harrow: based on the Healthy London Partnership agreed between members of the Healthy London asthma leadership group^8^

General standards:	• All CYP prescribed more than 6 short acting bronchodilator reliever inhalers (SABAs) in the previous year should also be prescribed inhaled corticosteroids (or another preventer drug) • All CYP with asthma should have evidence of being provided a Personal Asthma Action Plan (detailing medication administration, trigger factors and their avoidance, identification of danger signs of attacks, and what to do when these occur) • All CYP over 5 years should have a record of their best Peak Expiratory Flow • All CYP prescribed inhalers must have evidence in their records of having their inhaler technique assessed
During attacks:	• All CYP should have a measurement of oxygen saturation, repeated after treatment if abnormal. • All CYP over age 5 years should have a measurement of Peak Expiratory Flow (to include one after first dose of bronchodilator treatment to assess whether treatment was successful)
After treatment of the attack:	• All CYP prescribed oral corticosteroids should be reviewed within 2 working days of starting treatment with oral corticosteroids. • This review should include checking inhaler technique, whether a Personal Asthma Action Plan has been issued or needs modifying, and what triggered the attack • The review should also include optimization of treatment • Oral Corticosteroids should be continued until the attack has resolved (as determined by the health professional) (BTS/SIGN)^6^

### Implications for future research, policy and practice

Medical audit, where clinicians reflect on their care provided for patients is a valuable method for learning, self-reflection and driving change. By encouraging active discussion with colleagues, self-analysis and reflection on their care compared with evidence based practice, clinicians are able to identify areas for improvement and in the case of asthma attacks, can take appropriate action to reduce future risk. In this audit, of patients who had had asthma attacks, wherever they were treated, clinicians were able to identify possible preventable factors and generate ideas and action for optimization of asthma care by dealing with modifiable risks; with the result that further attacks were prevented. Other methods for promoting self-analysis and reflection on by groups of colleagues, of care provided, should also be encouraged; for example by conducting a significant event analysis for any asthma attack and death.

While clinicians’ asthma knowledge and optimization of care cannot be automated, the identification of patients at risk could be improved using technology. One of the recommendations by the NRAD was to encourage general practice software companies to develop systems for identifying patients at risk, particularly regarding prescribing issues. To date this has not been satisfactorily implemented in our view. However, a new exciting development in London may help. The Whole Systems Integrated Care project in eight North West London CCGs including Harrow, has developed a dynamic data driven ‘Asthma Radar’ launched in November 2017.^7^ This is a dashboard of practice and secondary care patient level data extracted weekly, and based on derived ‘Red Flag’ variables (such as asthma attacks and admissions, excess reliever and insufficient preventer prescriptions, poor inhaler technique and a number of others) together with risk factors for asthma attacks such as obesity, raised eosinophyls, and co-morbid conditions, (Tables 2–2 GINA,^8^ and Table 11 BTS/SIGN 153^9^) which enables clinicians to easily identify patients with Red Flags or risk factors predisposing to asthma attacks for priority optimization of care. Reports on the effectiveness of this system will be published in due course.

## Conclusions

This Harrow audit has provided an example of how clinicians can focus learning from patients who have had asthma attacks and utilize these events as a catalyst for active reflection in particular on modifiable risk factors. Through identification of these risks and active optimization of management, preventable asthma attacks could become ‘never events’.

## Methods

All general practices (*n* = 34) in Harrow were invited to opt-in to a local Quality improvement LIS aimed at reducing admissions in CYP by 10% in the following year. Participating practices were paid an incentive, if they actively contributed to a retrospective medical record audit, and participated in peer group discussions and agreed to implement subsequent recommendations to improve patient care. Harrow practices care for about 62,000 CYP (average 1850 per practice, range 450–5500 CYP). Subjects: CYP in the practices, aged 0–19 (i.e., <19 years), whose medical records indicated they had an asthma attack during 1 March 2015–30 September 2015; i.e., those who had been treated for asthma or wheezy attacks: in Accident and Emergency departments; or in the UCC; or in the GP practice. Patients were identified in July 2016, through computer record searches, and checking through correspondence during the 6 month audit period. Practices were also asked to do a prospective audit of patients who were subsequently treated for attacks.

The agreed standards for the audit (see Table 1) were developed on the basis of the NRAD findings and recommendations,^1^ UK National^9^ and International strategy documents,^8^ and in consultation with colleagues from the group that developed the London asthma standards for children and young people.^10^

Data: Data was extracted manually by practice staff from the medical records, annonymised but identifiable to the participating practice, and then entered online using Typeform software.^11^ The methodology is further detailed online (http://bit.ly/1Snt9Dd) and the data collection form used by practices is included in Appendix 1. Audit data was downloaded (in Excel format) from Typeform, then analysed and presented to practices, Data was analysed manually, summarized and presented to practices by Dr Mark Levy. Assessment of attainment of the LIS (10% reduction in urgent hospital admissions) for the purpose of paying the CCG, was done by the CCG based on NHS England SUS data.

The audit dataset (with practice ID anonymised) is available from the author on request.

## Electronic supplementary material

Appendices 1-3
