# Supplementary material for: Management of children and young people (CYP) with asthma: a clinical audit report
Source: NPJ Prim Care Respir Med. 2018 May 21;28:16. doi: 10.1038/s41533-018-0087-5 (PMC5962615; doi:10.1038/s41533-018-0087-5)
Supplement: Supplementary file 1 — Appendices 1-3 [file 41533_2018_87_MOESM1_ESM.pdf]

APPENDIX 1: Childhood Asthma Audit: © Dr Mark L Levy Ver 6: 11.3.2015

Please see Harrow Childhood Asthma Audit – Background and instructions for completion

CCG/ Health Board **Practice Code(UK):** For non UK create a personal code (eg_dialing code+name)

Choose for this patient: ☐ **Baseline Audit (attacks during previous year) or** ☐ **Prospective Audit**

Patient initials: **Confidential Identifier:** **Male:**☐ **Female:** ☐
Age to nearest year **Weight (Kg):** **Height (cm):** **BMI:**

Attack Date: / /
Where treated : Practice ☐ **Hospital inpatient** ☐ **WalkinCentre/UCC** ☐ **A&E/ED** ☐
(Tick which apply)
Treated by(Tick which apply): Doctor ☐ **Nurse** ☐ **Respratory trained Nurse** ☐ **Nurse Practtioner** ☐
Discharge date (if admitted): / /

Asthma treatment at the time of (ie just before) attack (From your records):
Reliever Inhaer (Short acting) dose mcg frequency device Not Known ☐
Preventer Inhaler dose mcg frequency device Not Known ☐
Combination Inhaler dose mcg frequency device Not Known ☐
(Preventer/Reliever)
Long acting Reliever drug : Salmeterol ☐ Formoterol ☐ Theophylline ☐ Other ☐
LTRA tablet: Yes ☐ No ☐ Not Known
Spacer : Yes ☐ No ☐ Not Known

In the 12 months before attack:
How many Short Acting reliever inhalers prescribed (salbutamol/terbutaline)? / Not Known ☐
How many Short Acting reliever inhalers bought OTC (salbutamol/terbutaline)? /Not Known ☐
How many preventer inhalers prescribed (named above)? / Not Known ☐

Personal Asthma Action Plan: Issued ☐ Never Issued: ☐ Date Issued : / /
Date last revised: / / Not Known ☐
Was an asthma review done in the 12 months before the attack: Yes ☐ No ☐ Not Known
Date reviewed / / Was current asthma symptom control assessed ?
Yes☐ No ☐ Not Known ☐
If Yes How: ACT ☐ ACQ ☐ RCP 3Qs ☐ GINA ☐ Other ☐ Not Known ☐)
If yes - was his/her asthma : well controlled ☐ Poorly controlled ☐ Not Known ☐
inhaler technique checked? Yes ☐ If Yes (DateLastChecked: / /) No ☐ Not Known ☐
Was it poor ☐ ** or good ☐ ? If Poor -> Inhaler technique corrected ☐ new inhaler Pxed ☐ NoAction ☐
Is there a record of previous best peak flow? Yes ☐ No ☐ Not Known ☐ if yes: l/Min

During the attack:
Any Risk Factors identified: None identified ☐Previous admission ☐ Previous A&E ☐ or (P)ICU ☐
Previous Life threatening attack ☐ Smoker ☐ Passive smoker ☐Obesity ☐ Food Allergy ☐
Poor Inhaler technique ☐ Low FEV1 ☐ > 3 asthma drug types ☐ Poor Control ☐ Other
Was Peak Flow measured? Yes ☐ No ☐ Not Known ☐
if yes l/Min Before treatment ; l/Min After treatment
Was oxygen saturation measured? Yes ☐ No ☐ Not Known ☐
if yes % Before treatment ; % After treatment or not done after treatment ☐
Was Salbutamol Given? Given but route not known ☐ By Nebuliser ☐ By Nebuliser with oxygen ☐
Multiple puffs by Spacer ☐ Multiple puffs by pMDI inhaler ☐ Not Given ☐
Ipatropium bromide: Yes ☐ No ☐ Not Known ☐
Was oxygen given ?: Yes ☐ No ☐ Not Known ☐

Were Oral corticosteroids prescribed: Yes ☐ No ☐ Not Known ☐
If yes: Prednisolone daily dose mg Not Known ☐
Duration: 3 Days ☐ 5 Days ☐ 7 Days ☐ until resolved ☐ Not Known ☐
How long did this attack take to resolve? Days (from treatment date) **leave blank if Not Known**
Post attack review: Not reviewed ☐ Date reviewed after attack / /

Reflection: ……………………………………………………………………………..
Why do you think this person had the attack? Ie what brought this about?
i) A Trigger Yes ☐ No ☐ Not Known ☐
If Yes was this due to: Pollen (tree/grass) ☐ **Exercise** ☐ **Infection** ☐ **Animal (cat,dog,horse)** ☐
Food ☐ **Other** ☐ please detail
ii) Medication: Not prescribed preventer ☐ Excess Reliever ☐ Betablocker ☐ NSAID ☐
other
iii) Non Adherence by patient/carer ☐
iv) Failure to recognise risk: by health professional ☐ or by patient or Carer ☐
Other : ☐ please detail:
What could have been done to prevent the attack?

Appendix 2: Examples of individualized feedback provided for the practices:

Practices were provided with a report detailing the results of their audited patients compared with those of Harrow overall, including specific comments about care of individual patients for whom the practice provided annonymised identification for the purpose of analysis and feedback. Two examples form the report to one of the practices are:

1. **Comment for one practice regarding prescription of inhaled corticosteroids for patients prescribed more than 6 SABA inhalers in the previous year:**

   'While you have done well to ensure these patients are prescribed inhaled corticosteroids - the fact that despite this they still remain uncontrolled (having attacks, being prescribed excess reliever medication) indicates a need for detailed review to decide on changing inhaler if poor technique, increased potency of inhaled corticosteroid (see Tables 9 and 10 in the new SIGN 153 Asthma Guideline)'

2. **Comment for one practice regarding individual patient:**

   'One child had 3 attacks in the last year (EMIS Number 56781) - this child is uncontrolled despite being on 100 mcg Fluticasone Bd (ie equivalent to 400 mcg beclometasone a day) He has had 10 prescriptions of salbutamol, has poor inhaler technique. despite having a spacer. I suggest urgent review and consider referral to paediatric respiratory specialist urgently.'

Appendix 3:

Results for one of the practices that repeated the audit against standards set for the Harrow CCG Audit of care (see Table 1). The results for the baseline audit (33 patients had 49 attacks) are compared with the subsequent audit of 19 patients each having one attack.

	**Baseline Audit**	**Re-audit**
Numbers of *CYP patients/attacks	33/49	19
**Before attacks**		
Number of patients prescribed more than 6 short acting bronchodilator reliever inhalers (**SABAs) in the previous year	9	1
Evidence of being provided a Personal Asthma Action Plan	10/33	17/19
Record of best Peak Expiratory Flow (> 5 years old)	8 of 21(38%)	10 of 15 (66%)
Inhaler technique assessed	5 of 33 (15%) patients prescribed inhalers	10 of 18 (55%) patients prescribed inhalers
**During attacks:**		
Oxygen saturation measured	17 of 49 attacks (34%)	10 of 19 attacks (52%)
Peak Expiratory Flow (> 5 years old)	9 of the 31elligible attacks (29%)	4 of the 15 elligible attacks (26%)
**After treatment of the attack:**		
Reviewed within 2 working days of starting treatment with oral corticosteroids.	2 of the attacks were followed up within 2 days (4%)<br><br>Most attacks were not followed up at all, and those that were ranged from 2 to 75 days	18 of the 19 attacks were followed up; 3 within 2 days, 11 under 10 days, and the other 5 up to 50 days after the attack.

* CYP = Children and Young People < 19 Years old
** SABA = Short Acting Bronchodilator Reliever (eg salbutamol)
